# A Novel Protozoa Parasite-Derived Protein Adjuvant Is Effective in Immunization with Cancer Cells to Activate the Cancer-Specific Protective Immunity and Inhibit the Cancer Growth in a Murine Model of Colorectal Cancer

**DOI:** 10.3390/cells13020111

**Published:** 2024-01-06

**Authors:** Rajesh Mani, Chloe G. Martin, Kanal E. Balu, Qingding Wang, Piotr Rychahou, Tadahide Izumi, B. Mark Evers, Yasuhiro Suzuki

**Affiliations:** 1 Department of Microbiology, Immunology and Molecular Genetics, University of Kentucky, Lexington, KY 40536, USA; rajesh.mani@uky.edu (R.M.);; 2 Department of Surgery, University of Kentucky, Lexington, KY 40536, USApiotr.rychahou@uky.edu (P.R.);; 3 Markey Cancer Center, University of Kentucky, Lexington, KY 40536, USA; 4 Department of Toxicology and Cancer Biology, University of Kentucky College of Medicine, Lexington, KY 40536, USA

**Keywords:** *Toxoplasma gondii*, protein adjuvant, cancer immunotherapy, CD8^+^ T cells, cytotoxic activity, IFN-γ, intratumoral T cells

## Abstract

Cancer-specific CD8^+^ cytotoxic T cells play important roles in preventing cancer growth, and IFN-γ, in addition to IL-12 and type I interferon, is critical for activating CD8^+^ cytotoxic T cells. We recently identified the capability of the amino-terminus region of dense granule protein 6 (GRA6Nt) of *Toxoplasma gondii*, an intracellular protozoan parasite, to activate IFN-γ production of microglia, a tissue-resident macrophage population. Therefore, in the present study, we examined whether recombinant GRA6Nt protein (rGRA6Nt) functions as an effective adjuvant to potently activate cancer-specific protective immunity using a murine model of MC38 colorectal cancer (CRC). When mice were immunized with non-replicable (either treated with mitomycin C or irradiated by X-ray) MC38 CRC cells in combination with rGRA6Nt adjuvant and received a challenge implantation of replication-capable MC38 tumor cells, those mice markedly inhibited the growth of the implanted tumors in association with a two-fold increase in CD8^+^ T cell density within the tumors. In addition, CD8^+^ T cells of the immunized mice secreted significantly increased amounts of granzyme B, a key mediator of the cytotoxic activity of CD8^+^ T cells, and IFN-γ in response to MC38 CRC cells in vitro when compared to the T cells from unimmunized mice. Notably, the protective effects of the immunization were specific to MC38 CRC cells, as the immunized mice did not exhibit a significantly inhibited growth of EL4 lymphoma tumors. These results indicate that rGRA6Nt is a novel and effective protein adjuvant when used in immunizations with non-replicable cancer cells to potently activate the protective immunity specifically against the cancer cells employed in the immunization.

## 1. Introduction

The recent development of the cancer immunotherapy with immune checkpoint inhibitors has made notable progress in cancer therapy [1,2,3,4]. However, since the effects of the immune checkpoint inhibitors are not specific to anti-cancer immunity, this therapy often causes unwanted overly stimulated immune responses to unrelated antigens and causes potentially serious toxic side effects [5,6]. In addition, this new therapy has shown only limited efficacy in the treatment of some of the more prevalent cancers, including colorectal cancers (CRC) [1,2,3,4]. Chimeric antigen receptor (CAR) T cell therapy is another recent advancement in cancer immunotherapy. CAR T cells express genetically modified antigen receptors composed of the antigen-specific binding component of a monoclonal antibody combined with T cell receptor signaling molecules required for T cell activation [7]. Although this treatment is specific to a single-target antigen and quite effective for B cell lymphomas that commonly express CD19 [7], applying this method to treat other types of cancers is challenging because of variations of cancer antigens among different cancer types and individual patients. Thus, it is critical to develop a new method that can potently activate anti-cancer protective immunity specifically with respect to various types of cancers in different individuals.

A valuable material to overcome the variation in antigens among cancer patients will be the use of their own cancer cells for immunization. However, since cancer-specific antigens are often not strongly immunogenic, we need to employ a potent immunostimulant to efficiently activate cancer-specific protective T cells in the immunization. *Mycobacterium bovis* bacillus Calmette-Guerin (BCG) has been clinically used as an immunostimulant for treating bladder cancer and metastatic melanoma [8,9]. An important effect of BCG is an induction of maturation of dendritic cells (DC) [10,11], which are the professional antigen-presenting cells of the immune system. Thus, BCG most likely functions as an adjuvant to activate the protective T cell immunity against cancer-specific antigens through an induction of DC maturation. This point is supported by the recent evidence that BCG therapy activates cancer-specific CD4^+^ T cells that produce IFN-γ in a murine model of bladder cancer [12]. *Toxoplasma gondii*, an intracellular protozoan parasite, has a superior activity to induce maturation and accumulation of DC [13,14]. We previously compared anti-cancer activity of the immune responses induced by *T. gondii* with those induced by BCG using subcutaneously implanted Lewis lung carcinoma (LLC) and EL4 lymphoma [15,16]. Our studies demonstrated that the anti-cancer effects of the immune responses induced by *T. gondii* are much greater than those induced by BCG against both of these cancers [15,16]. Recent studies by others using vaccine strains, either non-replicable in vivo [17,18,19,20] or a virulent [21], further demonstrated that *T. gondii* is an efficient immunostimulant to activate anti-cancer protective immunity against various types of cancers including ovarian cancer and melanoma using murine models.

Our recent study revealed that an immunization with recombinant protein of the amino-terminus region of dense granule protein 6 (rGRA6Nt) of *T. gondii* without the use of any adjuvant potently activates CD8^+^ cytotoxic T cells against this molecule [22]. Our recent study also identified that rGRA6Nt activates IFN-γ production by microglia, tissue resident macrophages in the brain parenchyma [23]. IFN-γ has been shown to enhance cytotoxic T lymphocytes and NK cell activities [17,24,25]. Therefore, it would be possible that rGRA6Nt has a potent adjuvant activity to facilitate an activation of CD8^+^ cytotoxic T cells to target antigens. Notably, the presence of tumor-infiltrating CD8^+^ T cells is an indicator of positive prognosis in cancer patients [26,27]. Thus, in the present study, we examined an adjuvant effect of rGRA6Nt in cancer immunotherapy and discovered that an immunization of mice with non-replicable MC38 CRC cells, which were pretreated with mitomycin C (MMC) or a lethal dose of ionizing radiation, in combination with rGRA6Nt adjuvant strongly activates the MC38-specific protective immunity against a challenge implantation of the tumor cells, and that the protective effect is associated with significantly increased infiltration of CD8^+^ T cells into the MC38 tumors and increased activity of CD8^+^ T cells to secrete granzyme B, a key cytotoxic molecule, and IFN-γ in response to the cancer cells. 

## 2. Materials and Methods

### 2.1. Mice 

C57BL/6 mice were from the Jackson Laboratory (Bar Harbor, ME, USA). Female mice were used for all studies. There were 5 or 6 mice in each experimental group in each experiment. The studies were performed in accordance with approved protocols from the Institutional Animal Care and Use Committee. 

### 2.2. Tumor Cell Lines

MC38 CRC cell line was obtained from Kerafast (Boston, MA, USA) and maintained in Dulbecco’s modified MEM (DMEM) with 10% fetal bovine serum (FBS), 2 mM L-glutamine, 0.1 mM nonessential amino acids, 1 mM sodium pyruvate, and 10 mM HEPES. All of these reagents for the culture were from Gibco/ThermoFisher Scientific (Waltham, MA, USA). The cell line was passaged every 3 or 4 days to maintain it within a range of approximately below 80% confluent. EL4 lymphoma cell line was from ATCC (Manassas, VA, USA) and maintained in DMEM with 10% FBS and penicillin/streptomycin. This cell line was also passaged every 3–4 days by following the company’s instruction. 

### 2.3. Immunization with Non-Replicable MC38 CRC Cells 

MC38 CRC cells were treated with MMC (MilliporeSigma, Burlington, MA, USA) or irradiated with 30 Gy X-ray to make them non-replicable for immunizations. For MMC treatment, MC38 cells were suspended in DMEM with 10% FBS, 2 mM-L-glutamine, 0.1 mM of nonessential amino acids, 1 mM sodium pyruvate, and 10 mM HEPES at 5 × 10^6^ cells/mL and incubated with MMC (100 μg/mL) for 1 h. Thereafter, cells were washed and suspended in Dulbecco’s modified PBS (DPBS) (Gibco). Mice were immunized intraperitoneally with 1 × 10^6^ of the MMC-treated MC38 CRC cells with either 10, 20, or 40 μg of rGRA6Nt adjuvant twice with a four-week interval. The rGRA6Nt was produced and purified at the Protein Core Laboratory at our college using the plasmid kindly provided by Drs. Corinne Mercier and Marie-France Delauw of the University of Grenoble, France. As a control, one group of mice was immunized with MMC-treated MC38 cells alone without any adjuvant. An additional group of mice was treated with DPBS as a negative control. For the treatment with irradiation, MC38 cells suspended in HBSS with 2% FBS at 1 × 10^6^ cells/mL were irradiated with 30 Gy by X-ray with PXI XRAD 225XL with the aluminum filter (Precision X-ray, Madison, CT, USA) at an approximate rate of 2.2 Gy/min at the X-ray Service Center of the Department of Toxicology and Cancer Biology of the University of Kentucky College of Medicine [28,29]. Mice were immunized with the irradiated MC38 CRC cells in the same manner as the MMC-treated MC38 cells. The incapability of both the MMC-treated and irradiated MC38 cells to replicate was confirmed by culturing these cells for 2–3 days.

### 2.4. Challenge Implantation of MC38 CRC and EL4 Lymphoma Cells 

Two weeks after the second immunization with MMC-treated or irradiated MC38 CRC cells, mice were injected subcutaneously with 1 × 10^6^ or 5 × 10^5^ MC38 cells in 0.1 mL of DPBS. In one experiment, the immunized mice were injected subcutaneously with 5 × 10^5^ EL4 lymphoma cells in the same manner. Sizes of the tumors were measured every 3–4 days using digital caliper. When the diameter of the tumor reached 15 mm, mice were euthanized for humane reasons and time of death was recorded. 

### 2.5. Immunohistochemical Staining for CD4^+^ and CD8^+^ T Cells That Infiltrated into MC38 Tumors

MC38 tumors grown in mice immunized with MMC-treated MC38 cells with 40 μg of rGRA6Nt adjuvant and unimmunized mice were surgically resected on Day 8 and Day 11 after an implantation of the tumor cells. Those tumors were cut into halves, and half of each tumor was fixed in a solution containing 10% formalin, 5% acetic acid, and 70% ethanol. The fixed tumors were embedded in paraffin, and sections (4 μm thickness) of the paraffin-embedded tumors were stained with anti-mouse CD4 or anti-mouse CD8α rabbit monoclonal antibody (Cell Signaling Technology, Danvers, MA, USA) using Ventana Discovery Ultra instrument (Roche Diagnostics, Indianapolis, IN, USA). The staining for CD8α was as follows: After deparaffinization and antigen retrieval with Ventana CC1 (Roche), the slides were incubated with anti-CD8α antibody at 1:250 dilution at 37°C for 1 h. The slides were then incubated with Ventana anti-rabbit-HQ (Roche) for 20 min, followed by incubation with Ventana anti-HQ-HRP (Roche). The staining was then amplified using Ventana’s Discovery TSA Amplification Kit (Roche) for 16 min, followed by linking with Discovery Amplification Multimer-HRP (Roche) for 20 min and DAB detection. Slides were counterstained with Meyer’s hematoxylin, blued, and mounted. Staining for CD4 was performed in the same manner with 1:50 dilution of anti-CD4 antibody.

### 2.6. Counting Numbers of CD4^+^ and CD8^+^ T Cells That Had Infiltrated into the MC38 Tumors 

Numbers of CD4^+^ and CD8^+^ T cells that had infiltrated into the tumors were counted microscopically by scanning each section of MC 38 tumors at 200× magnification from one end to the other end of the tumor sections at three different locations (approximately one forth from the top of the section, the middle of the section, and three forth from the top of the section). When the tumors were too small to measure the T cell numbers to scan at the three different locations described above, the scanning was performed at only the middle of the section or one third and two thirds from the top of the section. The densities of CD4^+^ and CD8^+^ T cells within the tumors were indicated as per mm^2^ of each tumor. 

### 2.7. Purification of CD4^+^ and CD8^+^ T Cells and Their Cultures with MC38 CRC Cells 

CD4^+^ and CD8^+^ T cells were purified from the spleens of the mice immunized with MMC-treated MC38 CRC cells with 40 μg of rGRA6Nt adjuvant at 2 weeks after the second immunization using anti-CD4 and anti-CD8 antibody-coated micromagnetic beads using MACS system (Miltenyi Biotech, Gaithersburg, MD, USA) [22,30]. We routinely purify these T cells using this method, and the purity of these CD4^+^ and CD8^+^ T cells with this purification method is consistently >95%. The purified T cells were suspended in RPMI1640 medium (MilliporeSigma) containing 10% FBS (Cytiva, Vancouver, BC, USA) and 2 mM L-glutamine and cultured in 96-well culture plates at 5 × 10^5^ cells/well with and without MMC-treated MC38 CRC cells at 1 × 10^5^ cells/well for 72 h. As a control, CD4^+^ and CD8^+^ T cells from unimmunized mice were cultured with and without MC38 cells in the same manner. The concentration of GzmB and IFN-γ in their culture supernatants were measured using commercial kits from R&D Biosystems (Mineapolis, MN, USA) and Meso Scale Discovery (Rocksville, MD, USA), respectively, by following manufactures’ manuals. 

### 2.8. Statistical Analysis

Levels of significance in differences between experimental groups were determined by Student’s *t* or Mann–Whitney *U* test using GraphPad Prism software 9.0. Log-rank (Mantel-Cox) test (GraphPad Prism) was used for determining levels of significance in differences in the survival curves of mice between experimental groups. Differences that had *p* values <0.05 were considered significant. 

## 3. Results

### 3.1. Immunization with MMC-Treated MC38 CRC Cells with rGRA6Nt Adjuvant Confers Potent Protections against a Challenge Implantation of This Cancer Cells in a Dose-Dependent Manner of the Adjuvant

**Figure 1:**
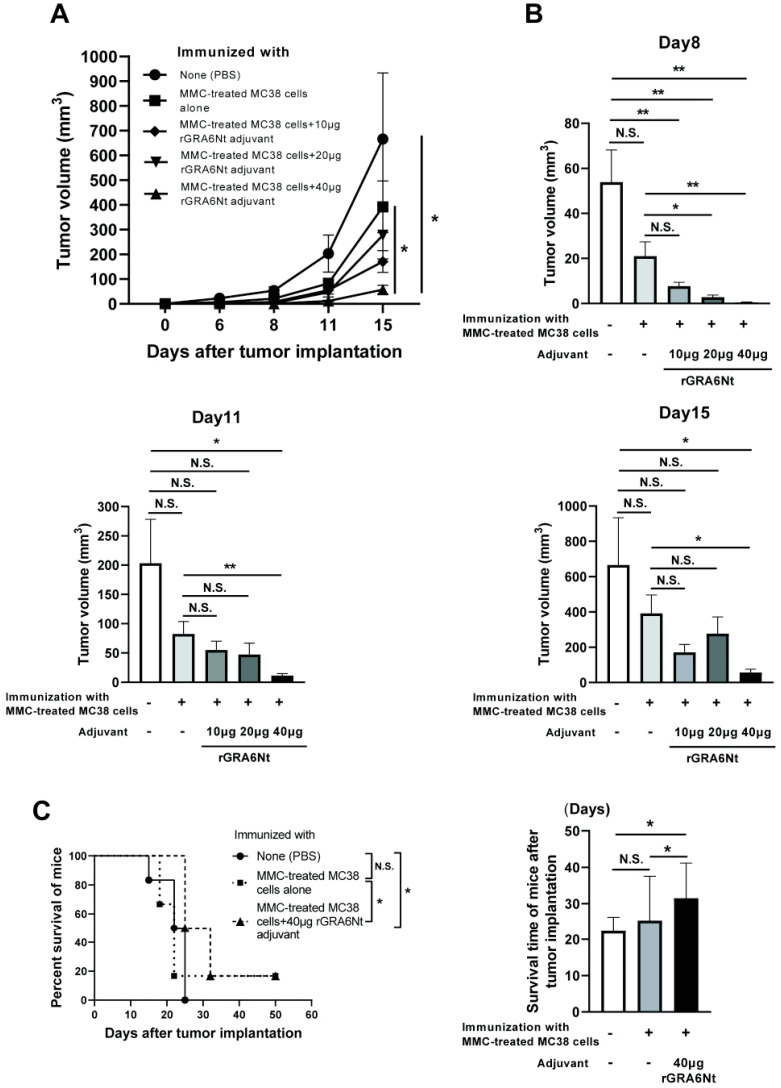
Immunizations with MMC-treated MC38 CRC cells plus 40 μg of rGRA6Nt adjuvant markedly inhibit growth of MC38 tumors after its challenge implantation. C57BL/6 mice were immunized intraperitoneally with 1 × 10^6^ cells of MMC-treated MC38 cells with 10, 20, or 40 μg of rGRA6Nt adjuvant twice with a 4-week interval. As a control, mice were immunized with the MMC-treated MC38 cells without any adjuvant in the same manner. An additional group of mice was immunized with PBS as a negative control. Two weeks after the second immunization, mice were challenged with a subcutaneous implantation of 1 × 10^6^ cells of replication-capable MC38 CRC cells. Sizes of tumors grown from the challenge implantation were measured on Days 5, 8, 11, and 15 after the implantation. (**A**) The time course of growth of the tumors in each experimental group. (**B**) The volumes of tumors in each experimental group on Days 8, 11, and 15 after the challenge implantation. (**C**) The mean survival times and survival curves of unimmunized mice and those immunized with either MMC-treated MC38 cells alone or those cancer cells with 40 μg of rGRA6Nt adjuvant after a challenge implantation of MC38 CRC cells. There were six mice in each experimental group. * *p* < 0.05 and ** *p* < 0.01. N.S., Not significant.

C57BL/6 mice were immunized intraperitoneally with 1 × 10^6^ cells of MMC-treated MC38 CRC cells with either 10, 20, or 40 μg of rGRA6Nt adjuvant twice within a four-week interval. As a control, one group of mice received immunization with the MMC-treated MC38 cells without any adjuvant. Another group of mice were injected only with DPBS as unimmunized mice. Two weeks after the second immunization, mice were challenged with a subcutaneous injection of 1 × 10^6^ cells of MC38 CRC at the right flank. The time courses of growth of the implanted tumors in each of the experimental groups during 15 days after the tumor implantation are shown in Figure 1A. On Day 15 after the tumor implantation, the majority of mice in the unimmunized control group were euthanized because their tumor sizes reached 15 mm in their diameter. The tumor sizes of experimental groups on Days 8, 11, and 15 after the tumor implantation are also displayed individually in Figure 1B. 

On Day 6, small solid tumors were detected only in the two groups, unimmunized or immunized with the tumor cells without rGRA6Nt adjuvant. On Day 8, solid tumors became detectable in almost all of mice except the group immunized with MMC-treated MC38 cells plus 40 μg of rGRA6Nt adjuvant, in which the tumor was not detectable in two of six mice. At this time, the tumor volumes in each of the immunized groups with either 10, 20, or 40 μg of rGRA6Nt adjuvant were significantly smaller than those in the control mice with no immunization (*p* < 0.01, Figure 1B). Furthermore, the tumor volumes in the immunized group with 20 or 40 μg of rGRA6Nt adjuvant were also significantly smaller than those of the mice immunized with only the tumor cells with no adjuvant (*p* < 0.05 for 20 μg of rGRA6Nt adjuvant, and *p* < 0.01 for 40 μg of the adjuvant, Figure 1B). On both Day 11 and Day 15, tumor volumes of only mice immunized with the tumor cells with 40 μg of rGRA6Nt adjuvant remained significantly smaller than those of the unimmunized controls (*p* < 0.05, Figure 1B) and those of the mice immunized with no adjuvant (*p* < 0.01 on Day 11 and *p* < 0.05 on Day 15, Figure 1B). Notably, the average volumes of the tumors in the mice immunized with 40 μg of rGRA6Nt adjuvant were 18 times and 12 times smaller than those of the unimmunized mice on Day 11 and Day 15, respectively (Figure 1B). In addition, the tumor volumes of the former were nine times and seven times less than those of the mice immunized with no adjuvant (*p* < 0.01 on Day 11 and *p* < 0.05 on Day 15, Figure 1B). Consistent with the marked suppression of the tumor growth in mice immunized with MMC-treated MC38 CRC cells with 40 μg of rGRA6Nt adjuvant, only this group of mice survived significantly longer than the unimmunized control mice and those immunized with only MMC-treated MC38 cells without any adjuvant (*p* < 0.05, Figure 1C). The mean survival time of mice immunized with the tumor cells with the rGRA6Nt adjuvant was also significantly longer than those of the unimmunized mice and those immunized with the cancer cells without any adjuvant (*p* < 0.05, Figure 1C). These results indicate that the immunization with MMC-treated MC38 CRC cells plus 40 μg of rGRA6Nt adjuvant is markedly effective to induce a potent protection against a challenge implantation with these tumor cells. 

### 3.2. Immunization with Irradiated MC38 CRC Cells with rGRA6Nt Adjuvant Confers Potent Protections against a Challenge Implantation of This Cancer Cells in a Dose-Dependent Manner of the Adjuvant

**Figure 2:**
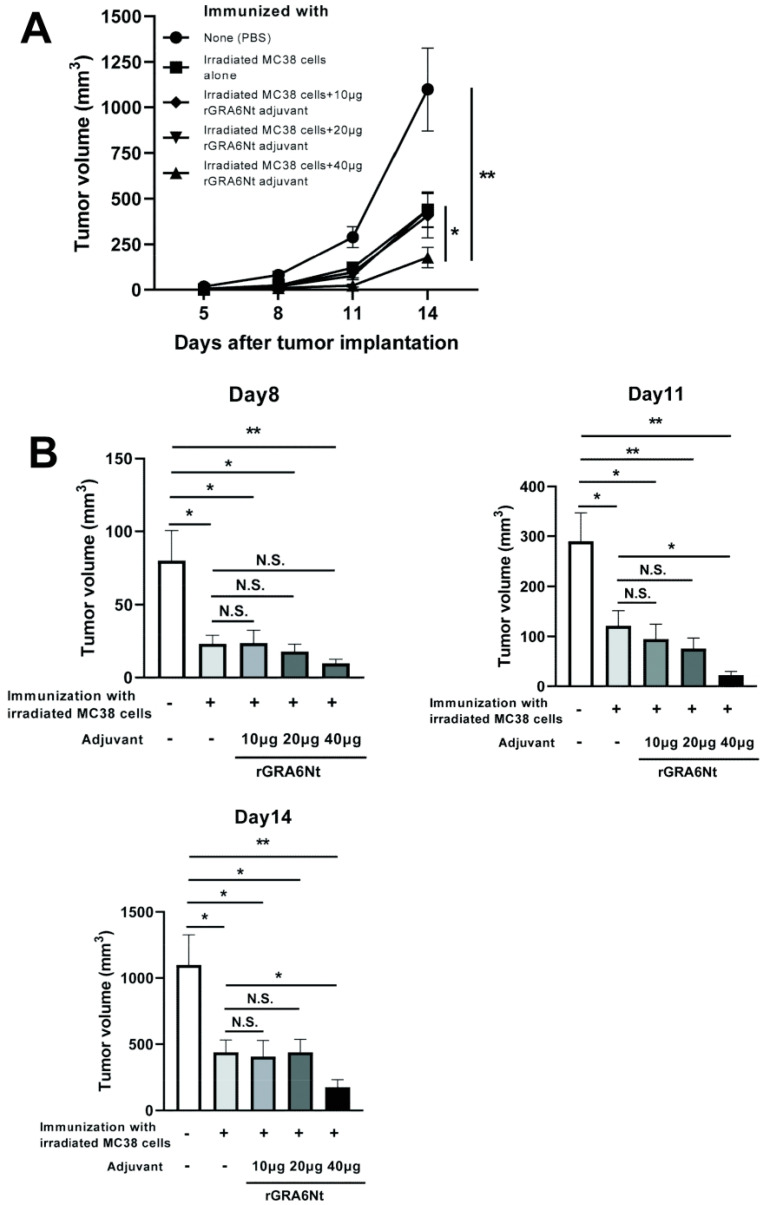
Immunizations with irradiated (30 Gy) MC38 CRC cells plus 40 μg of rGRA6Nt adjuvant markedly inhibit growth of MC38 tumors after its challenge implantation. C57BL/6 mice were immunized intraperitoneally with 1 × 10^6^ cells of the irradiated MC38 cells with 10, 20, or 40 μg of rGRA6Nt adjuvant twice with a 4-week interval. As a control, mice were immunized with the irradiated MC38 cells without any adjuvant in the same manner. An additional group of mice was immunized with PBS as a negative control. Two weeks after the second immunization, mice were challenged with a subcutaneous implantation of 1 × 10^6^ cells of replication-capable MC38 CRC cells. Sizes of tumors grown from the challenge implantation were measured on Days 5, 8, 11, and 14 after the tumor implantation. (**A**) The time course of growth of the tumors in each experimental group. (**B**) The volumes of tumors in each experimental group of on Days 8, 11, and 14 after the challenge implantation. There were five or six mice in each experimental group. * *p* < 0.05 and ** *p* < 0.01. N.S., Not significant.

We also performed immunizations with irradiated (30 Gy) MC38 CRC cells (1 × 10^6^ cells) with and without 10, 20, or 40 μg of rGRA6Nt adjuvant in the same way as the immunization with the MMC-treated MC38 cells and applied a challenge implantation of MC38 tumor cells. The time courses of growth of the implanted tumors are shown in Figure 2A. Consistent with the immunization with MMC-treated MC38 cells with rGRA6Nt adjuvant, immunizations with irradiated MC38 cells plus 40 μg of rGRA6Nt adjuvant conferred a potent protection against a challenge implantation with this tumor cells (Figure 2A). The average volumes of the tumors in this immunized group were 13 times and 6 times smaller than those of unimmunized control group on Day11 and Day 14, respectively (*p* < 0.01 for both time points, Figure 2B). In addition, the tumor volumes of the former were also 5.5 times and 2.5 times smaller than those of mice immunized with the tumor cells with no adjuvant (*p* < 0.05 for both time points, Figure 2B). A minor difference in the immunizations with the irradiated MC38 CRC cells from those with the MMC-treated cancer cells was that the immunizations with the former without any adjuvant provided a low but still significant inhibition of growth of MC38 tumors after its challenge implantation (*p* < 0.05, Figure 2B). A previous study demonstrated a better immunostimulatory activity of irradiated CT26 CRC cells than that of MMC-treated tumor cells [31]. There are also some studies reporting suppressive effects of low doses (10–20 μg/mL) of MMC on the immunostimulatory activities of dendritic cells [32,33]. However, even if these possible negative effects of MMC could have contributed to the slightly lower efficacy of the immunization with MMC-treated MC38 CRC cells without any adjuvant when compared to the immunization with irradiated MC38 cells without any adjuvant in the present study, the presence of rGRA6Nt adjuvant certainly overcame those suppressive effects of MMC and efficiently conferred a potent protection to suppress the growth of MC38 tumors. Overall, the results from the immunizations with both MMC-treated and irradiated MC38 CRC cells with different doses of rGRA6Nt adjuvant indicate that 40 μg of rGRA6Nt adjuvant is effective in immunizations with those non-replicable tumor cells to induce a potent protection against a challenge implantation of the MC38 tumors.

### 3.3. Immunizations with Non-Replicable MC38 CRC Cells with rGRA6Nt Adjuvant Induces Increased Infiltration of CD8^+^ T Cells into MC38 Tumors Implanted into These Mice 

**Figure 3:**
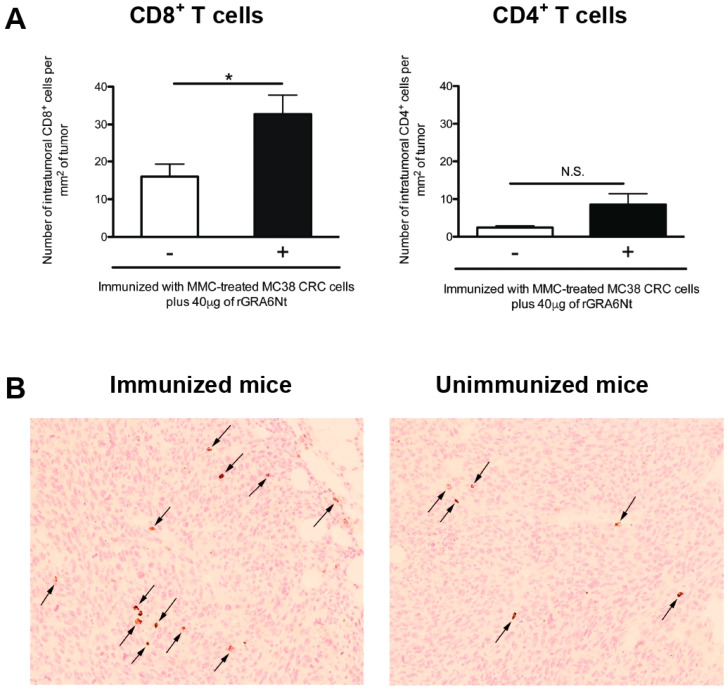
Immunizations with MMC-treated MC38 CRC cells plus 40 μg of rGRA6Nt adjuvant significantly increases the density of CD8^+^ T cells that infiltrate into implanted MC38 tumors. C57BL/6 mice were immunized intraperitoneally with 1 × 10^6^ cells of MMC-treated MC38 cells with 40 μg of rGRA6Nt adjuvant twice with a 4-week interval. As a control, mice were immunized with PBS as a negative control. Two weeks after the second immunization, mice were challenged with a subcutaneous implantation of 1 × 10^6^ cells of replication-capable MC38 CRC cells. MC38 tumors grown in these two groups of mice were surgically resected on Day 9, Day 11, and Day 15 after its challenge implantation, and those tumors were applied for immunohistochemical staining with anti-CD4 and anti-CD8α antibodies. The density of CD4^+^ and CD8^+^ T cells (numbers of those intratumoral T cells/mm^2^ of tumor) that had infiltrated into the tumors were counted microscopically by scanning each section of MC 38 tumors at 200× magnification from one end to the other end of the tumor sections at three different locations (approximately one fourth from the top of the section, the middle of the section, and three fourths from the top of the section). When the tumors are too small to measure the T cell numbers to scan at the three different locations described above, the scanning was performed at only the middle of the section or one third and two third from the top of the section. (**A**) The density of intratumoral CD4^+^ and CD8^+^ T cells in the tumors grown in the immunized and unimmunized mice. In the unimmunized control group, there were three mice on Day 9, one mouse on Day 11, and two mice on Day 15. In the immunized mice, there were three mice on Day 9, one mouse on Day 11, and one mouse on Day 15. The figure shows the data from all of these mice combined for each experimental group. (**B**) A representative image of CD8^+^ T cells detected within the tumors of the immunized and unimmunized mice on Day 9 after the implantation of the tumor cells. Arrows indicate CD8^+^ T cells detected. * *p* < 0.05. N.S., Not significant.

The presence of tumor-infiltrating CD8^+^ T cells is known as an indicator of positive prognosis of cancer patients [26,27], as mentioned earlier. Therefore, we examined whether the inhibition of MC38 tumor growth in the mice immunized with non-replicable MC38 CRC cells plus 40 μg of rGRA6Nt adjuvant is associated with increased infiltration of CD8^+^ T cells into the tumors. At 8 and 11 days after a challenge implantation of MC38 cells, the tumors grown in the immunized and unimmunized control mice were applied for immunohistological staining for CD4 and CD8α, and numbers of CD4^+^ and CD8^+^ T cells present within the tumors were microscopically counted. Notably, the density of CD8^+^ T cells detected within the tumors (CD8^+^ T cell numbers/mm^2^ of the tumors) were two-fold greater in the immunized than unimmunized mice (*p* < 0.05, Figure 3A). A representative image of CD8^+^ T cells detected within the tumors of each of these two groups is shown in Figure 3B. Notably, the density of CD4^+^ T cells detected within the tumors were much lower than those of CD8^+^ T cells in both of the immunized and unimmunized mice and those numbers did not differ between these two groups (Figure 3A). These results strongly suggest that CD8^+^ T cells play a key role in the protective immunity induced by the immunization with non-replicable MC38 CRC cells plus rGRA6Nt adjuvant to inhibit the growth of implanted MC38 tumors.

### 3.4. Immunization with Non-Replicable MC38 CRC Cells with rGRA6Nt Adjuvant Activates CD8^+^ Cytotoxic and IFN-γ-Producing T Cells against the Tumor Cells

**Figure 4:**
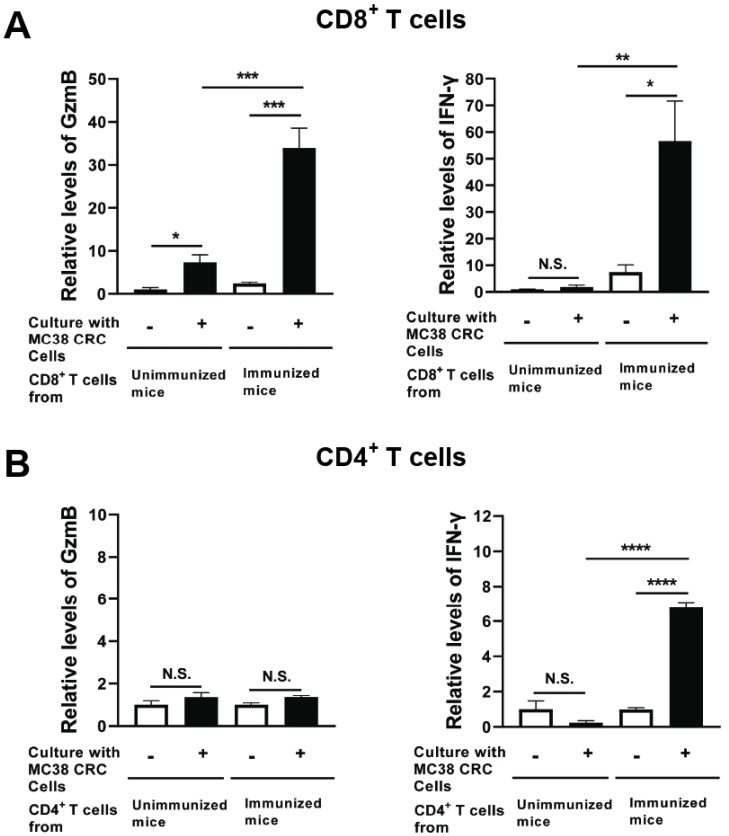
CD8^+^ T cells from mice immunized with MMC-treated MC38 CRC cells plus 40 μg of rGRA6Nt adjuvant secrete greater amounts of GzmB and IFN-γ in response to MMC-treated MC38 tumor cells in vitro. C57BL/6 mice were immunized intraperitoneally with 1 × 10^6^ cells of MMC-treated MC38 cells with 40 μg of rGRA6Nt adjuvant twice with a 4-week interval. Two weeks after the second immunization, CD4^+^ and CD8^+^ T cells were purified separately from their spleens (four mice) and pooled within each T cell population. Those T cell populations were then cultured (5 × 10^5^ cells/well) with and without the presence of MMC-treated MC38 cells (1 × 10^5^ cells/well) for 72 h. As a control, CD4^+^ and CD8^+^ T cells from unimmunized mice were purified and cultured with and without MC38 cells in the same manner. There were five wells in the cultures in each experimental group. The concentration of (**A**) GzmB and (**B**) IFN-γ in their culture supernatants were measured with ELISA using commercial kits. The levels of these effector molecules in the CD8^+^ T cell cultures are indicated as relative values to those detected in the supernatants of these T cells from unimmunized mice cultured without MC38 CRC cells. In case of CD4^+^ T cells, the effector molecule levels are indicated as relative values to those detected in the cultures of these T cells without MC38 CRC cells for each of immunized and unimmunized mouse groups due to high background values in the cultures without MC38 cells in the immunized mouse group. * *p* < 0.05, ** *p* < 0.01, *** *p* < 0.001, **** *p* < 0.0001. N.S., Not significant.

Two weeks after the second immunization with MMC-treated MC38 CRC cells plus 40 μg of rGRA6Nt adjuvant, CD4^+^ and CD8^+^ T cells were purified from their spleens and cultured with and without MMC-treated MC38 CRC cells for 72 h. As a control, CD4^+^ and CD8^+^ T cells from unimmunized mice were cultured in the same manner. The levels of GzmB, a key mediator of the cytotoxic activity of CD8^+^ T cells, in the culture supernatants of CD8^+^ T cells of the immunized mice were 14 times greater in their cultures with MC38 tumor cells than in their cultures without the tumor cells (*p* < 0.001, Figure 4A). In addition, the amounts of GzmB detected in the cultures of the immunized mouse CD8^+^ T cells with MC38 CRC cells were 4.8 times greater than those in the cultures of the unimmunized mouse T cells with the tumor cells (*p* < 0.001, Figure 4A), although the GzmB levels in the cultures of the latter T cells were significantly increased when the tumor cells were present in their cultures (*p* < 0.05, Figure 4A). 

IFN-γ levels in the supernatants of the immunized mouse CD8^+^ T cells cultured with MC38 CRC cells were also markedly greater than those in the cultures of those T cells without the tumor cells (*p* < 0.05, Figure 4A). In contrast, IFN-γ levels in the supernatants of unimmunized mouse CD8^+^ T cells did not differ between their cultures with and without MC38 CRC cells (Figure 4A). Furthermore, IFN-γ levels in the cultures of the immunized mouse CD8^+^ T cells with the tumor cells were approximately 40 times greater than those in the cultures of the unimmunized mouse T cells with the tumor cells (*p* < 0.01, Figure 4A). 

In the cultures of CD4^+^ T cells, GzmB levels in the cultures of those T cells from the immunized mice did not differ between their cultures with and without the tumor cells (Figure 4B). This is a clear contrast to CD8^+^ T cells of these mice described earlier, whereas IFN-γ levels in the supernatants of the immunized CD4^+^ T cells cultured with MC38 cells were greater than those detected in their cultures without the tumor cells (*p* < 0.0001, Figure 4B). These results together strongly suggest that the immunization with non-replicable MC38 CRC cells plus 40 μg of rGRA6Nt adjuvant efficiently activates both cytotoxic activity and IFN-γ production of CD8^+^ T cells, whereas this immunization activates only IFN-γ production of CD4^+^ T cells. 

### 3.5. The Protective Effects of Immunization with Non-Replicable MC38 CRC Cells with rGRA6Nt Adjuvant Are Specific to MC38 CRC

**Figure 5:**
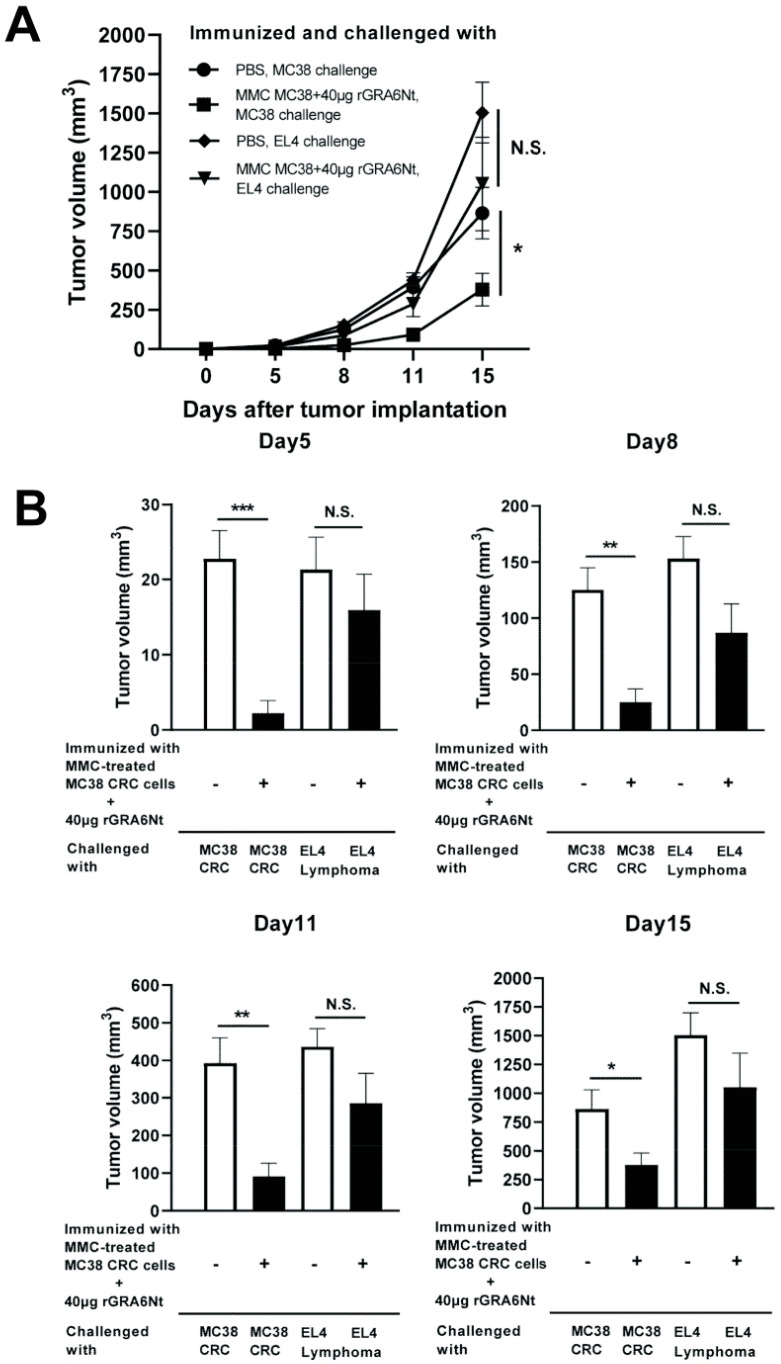
Immunizations with MMC-treated MC38 CRC cells plus 40 μg of rGRA6Nt adjuvant inhibit growth of MC38 tumors but not of EL4 lymphoma tumors after their challenge implantations. C57BL/6 mice were immunized intraperitoneally with 1 × 10^6^ cells of MMC-treated MC38 cells with 40 μg of rGRA6Nt adjuvant twice with a 4-week interval. Two weeks after the second immunization, mice were challenged with a subcutaneous implantation of 5 × 10^5^ cells of MC38 cells or EL4 lymphoma cells. As a control, mice immunized with PBS were challenged with these tumor cells in the same manner. Sizes of tumors grown from the challenge implantation were measured on Days 5, 8, 11, and 15 after the challenge implantation. (**A**) The time course of growth of the tumors in each experimental group. (**B**) The volumes of tumors at each experimental group of on Days 5, 8, 11, and 15 after the tumor implantation. There were six mice in each experimental group. * *p* < 0.05, ** *p* < 0.01, and *** *p* < 0.001. N.S., Not significant.

We examined whether the anti-cancer protective activity induced by the immunization with non-replicable tumor cells plus rGRA6Nt adjuvant is specific to the tumor cells used in the immunization. C57BL/6 mice were immunized with MMC-treated MC38 cells (1 × 10^6^ cells) plus 40 μg rGRA6Nt adjuvant twice and challenged subcutaneously with 5 × 10^5^ cells of either MC38 CRC or EL4 lymphoma cells. As a control, unimmunized mice were challenged with these tumor cells in the same manner. Tumor volumes of the immunized mice after a challenge implantation of MC38 cells were markedly smaller than those of unimmunized mice as expected (*p* < 0.001 for Day 5, *p* < 0.01 for each of Day 8 and Day 11, and *p* < 0.05 for Day 15, Figure 5A,B). In contrast, tumors of EL4 lymphoma grow quickly in both immunized and unimmunized mice in a similar manner, and those EL4 tumor volumes did not differ between the mice immunized with MC38 CRC cells and unimmunized mice (Figure 5A,B). These results indicate that the anti-cancer protective activity induced by the immunization with non-replicable MC38 cells with rGRA6Nt adjuvant is specific to these tumor cells.

## 4. Discussion

The present study revealed that rGRA6Nt, a protozoan parasite-derived protein molecule, has a potent adjuvant activity to efficiently activate anti-cancer protective immunity specifically against the cancer cells used in immunization with this adjuvant. The protective effects induced by immunizations with non-replicable (either treated with MMC or a lethal dose irradiation (30 Gy)) MC38 CRC cells with rGRA6Nt adjuvant against a challenge implantation of MC38 tumors were detected in a manner dependent on the doses of the adjuvant applied in the immunization. Among the three doses, i.e., 10, 20, and 40 μg of rGRA6Nt adjuvant used in the immunization, 40 μg of the adjuvant most effectively inhibited the growth of MC38 tumors after its challenge implantation. Our study also revealed that the immunization with non-replicable MC38 cells plus rGRA6Nt adjuvant did not confer any significant protections against a challenge implantation with EL4 lymphoma. These results indicate that rGRA6Nt is a novel, protozoan parasite-derived protein adjuvant effective to potently activate the protective immunity specifically against the cancer cells used for the immunization with this adjuvant. 

The present study also revealed that the anti-cancer protective effects induced by the immunization with non-replicable MC38 CRC cells with rGRA6Nt adjuvant is associated with markedly increased density of CD8^+^ T cells that have infiltrated into the MC38 tumors. This is a clear contrast to CD4^+^ T cells whose numbers detected within the tumors remained very low in both immunized and unimmunized mice, and no difference was detected in their densities within the tumors between these two groups. In addition, in vitro cultures of CD4^+^ and CD8^+^ T cells purified from the spleens of the immunized and unimmunized mice revealed significantly increased activities of CD8^+^ T cells from the immunized mice to secrete GzmB, a key cytotoxic molecule for the cytotoxic activity of the T cells and IFN-γ in response to MC38 tumor cells. It is well appreciated that cancer-specific CD8^+^ cytotoxic T cells can kill their target cancer cells [34], and the presence of tumor-infiltrating CD8^+^ T cells is an indicator of positive prognosis of cancer patients [26,27]. In contrast to CD8^+^ T cells, in response to MC38 tumor cells, CD4^+^ T cells of the immunized mice did not secrete any increased amounts of GzmB in response to the tumor cells when compared to those T cells of unimmunized mice T cells. Therefore, it is most likely that the significantly increased infiltration of CD8^+^ T cells into the tumors and the enhanced cytotoxic activity of these T cells in the mice immunized with non-replicable MC38 CRC cells plus rGRA6Nt adjuvant play a key protective role in the anti-cancer protective activity induced by the immunization. 

We recently discovered that CD8^+^ cytotoxic T cells against *T. gondii* cysts penetrate into the tissue cysts, which can grow into the sizes of over 100 μm in diameter within infected cells in a perforin-mediated manner for their destruction and elimination [30]. The present study revealed that the inhibition of MC38 tumors via immunization with non-replicable MC38 cells plus rGRA6Nt adjuvant is associated with markedly increased densities of CD8^+^ T cells that have infiltrated the tumors, as mentioned earlier. It is an intriguing possibility that those CD8^+^ T cells detected within the MC38 tumors have penetrated into the tumors using a mechanism similar to that which we observed in the penetration of CD8^+^ cytotoxic T cells into *T. gondii* cysts. It is also an intriguing possibility that rGRA8Nt is an effective adjuvant in activating those invasive CD8^+^ cytotoxic T cells specific to the tumor cells when this adjuvant is used in the immunization with the tumor cells. 

Increased IFN-γ production in response to MC38 CRC cells by CD8^+^ T cells of the mice immunized with non-replicable MC38 CRC cells plus rGRA6Nt adjuvant could also contribute to the potent protective effects of the immunization against a challenge implantation of MC38 tumors. It has been shown that MC38 CRC tumors contain large numbers of immunosuppressive tumor-associated macrophages (TAM) [35,36,37]. Converting these immunosuppressive TAM to M1-type pro-inflammatory macrophages can inhibit the tumor growth [35,38]. Notably, our study revealed that CD8^+^ T cells of the mice immunized with the MC38 CRC cells plus rGRA6Nt adjuvant secrete approximately 40-times greater amounts of IFN-γ in response to MC38 tumor cells than the T cells of the unimmunized control mice do, as shown in Figure 4. Therefore, when CD8^+^ T cells of the immunized mice infiltrate into the tumors and secret the large amounts of IFN-γ in response to the tumor cells, it is possible that the large amounts of secreted IFN-γ can spread into wide areas of the tumors and convert the immunosuppressive TAM to the pro-inflammatory M1-type macrophages and effectively suppress the tumor growth. 

Our previous studies demonstrated that the immune responses induced by *T. gondii* confer significantly greater protections against Lewis lung carcinoma and EL4 lymphoma tumors than do the immune responses induced by BCG [15,16]. BCG has been clinically used for treating bladder cancer and metastatic melanoma [8,9], as mentioned earlier. Recent studies by others also demonstrated that intralesional injections of a non-replicable *T. gondii* vaccine strain into established tumors induce potent protection against the cancer growth [17,19]. Another study also showed that intralesional injections of a less-virulent but proliferation-capable *T. gondii* strain into pre-implanted MC38 CRC tumors suppressed the tumor growth [21]. Therefore, there is the potential that rGRA6Nt, identified as a potent adjuvant in the present study, is a key mediator of the capability of *T. gondii* to potently activate anti-cancer immunotherapy. 

In a setting of surgically resectable CRC, the cancer cells prepared from the resected CRC could be treated with MMC or irradiation and mixed with rGRA6Nt for immunization to the patient, from whom the CRC was resected, to potently activate the protective immunity specific to his/her own cancer cells to prevent recurrence of the cancer in the patient. The results from the present study could confer a valuable basis for a new pathway with a novel rGRA6Nt adjuvant in this precision cancer immunotherapy approach. 

Unmethylated CpG deoxynucleotides (CpG ODN) [39,40,41], an agonist of TLR9 that recognize DNA with unmetylated CpG of bacterial and viruses, and polyincosinic-polycytidylic acid (Poly I:C) [42,43], an agonist of TLR3 that recognize the double-stranded RNA of viruses, have been used as an adjuvant to enhance the activation of T cells against cancer cells. The rGRA6Nt is a *T. gondii*-derived protein molecule and therefore unlikely to bind to either TLR3 or TLR9. Therefore, rGRA6Nt most likely displays its potent adjuvant activity through a mechanism(s) distinct from those of CpG ODN and Poly I:C. Thus, there is a notable potential that rGRA6Nt of *T. gondii* provides a new pathway in cancer immunotherapy to effectively activating anti-cancer protective immunity specifically to the cancer cells used in the immunization. 

## 5. Conclusions

This work demonstrated that the immunization of mice with non-replicable (treated with MMC or irradiated) MC38 CRC cells with a novel protozoan parasite-derived protein adjuvant, rGRA6Nt, potently activates the cancer cell-specific CD8^+^ cytotoxic T cells and IFN-γ-producing CD8+ T cells and inhibits the growth of tumors of the identical cancer cells after its challenge implantation. The tumor growth inhibition was associated with significantly increased numbers of CD8^+^ T cells infiltrated into the tumors. The specificity of the protective immunity was further confirmed by the evidence that the immunization with non-replicable MC38 cells plus rGRA6Nt adjuvant did not inhibit the growth of EL4 lymphoma tumors. The currently available adjuvants, CpG ODN and Poly I:C, for cancer immunotherapy are the ligand for the toll-like receptors that recognize microbial DNA and RNA. Since rGRA6Nt is a protein, this adjuvant most likely functions through a mechanism distinct from those of the currently available CpG ODN and Poly I:C adjuvants. Therefore, there is a potential that rGRA6Nt provides a new pathway in cancer immunotherapy by which to activate cancer cell-specific protective immunity to confer a notable protection specifically against the cancer cells used the immunization. 

## Data Availability

The data generated from this study are presented in this article. Further inquiries can be directed to the corresponding author.
